# Histopathological Examination of an Explanted Heart in a Long-Term Responder to Cardiac Stereotactic Body Radiotherapy (STereotactic Arrhythmia Radioablation)

**DOI:** 10.3389/fcvm.2022.919823

**Published:** 2022-07-07

**Authors:** Marcin Miszczyk, Mateusz Sajdok, Jerzy Nożyński, Magdalena Cybulska, Jacek Bednarek, Tomasz Jadczyk, Tomasz Latusek, Radoslaw Kurzelowski, Łukasz Dolla, Wojciech Wojakowski, Agnieszka Dyla, Michał Zembala, Anna Drzewiecka, Konrad Kaminiów, Anna Kozub, Ewa Chmielik, Aleksandra Grza̧dziel, Adam Bekman, Krzysztof Stanisław Gołba, Sławomir Blamek

**Affiliations:** ^1^IIIrd Radiotherapy and Chemotherapy Department, Maria Skłodowska-Curie National Research Institute of Oncology, Gliwice, Poland; ^2^Department of Electrocardiology, Upper Silesian Heart Center, Medical University of Silesia, Katowice, Poland; ^3^Department of Histopathology, Silesian Centre for Heart Diseases, Zabrze, Poland; ^4^Department of Electrocardiology and Heart Failure, Medical University of Silesia, Katowice, Poland; ^5^Department of Cardiology and Structural Heart Diseases, Medical University of Silesia, Katowice, Poland; ^6^Department of Electrocardiology, John Paul II Hospital, Kraków, Poland; ^7^Interventional Cardiac Electrophysiology Group, International Clinical Research Center, St. Anne’s University Hospital, Brno, Czechia; ^8^Department of Radiotherapy, Maria Skłodowska-Curie National Research Institute of Oncology, Gliwice, Poland; ^9^Radiotherapy Planning Department, Maria Skłodowska-Curie National Research Institute of Oncology, Gliwice, Poland; ^10^Department of Cardiac Surgery, Heart and Lung Transplantation, Mechanical Circulatory Support, Silesian Centre for Heart Diseases, Zabrze, Poland; ^11^Anaesthesiology and Intensive Care Unit, District Hospital in Oława, Oława, Poland; ^12^Department of Cardiac Surgery and Transplantology, Silesian Centre for Heart Diseases, Zabrze, Poland; ^13^Tumor Pathology Department, Maria Skłodowska-Curie National Research Institute of Oncology, Gliwice, Poland; ^14^Department of Medical Physics, Maria Skłodowska-Curie National Research Institute of Oncology, Gliwice, Poland

**Keywords:** ventricular tachycardia, structural heart disease, STAR, radioablation, stereotactic body radiotherapy (SBRT)

## Abstract

Cardiac stereotactic body radiotherapy is an emerging treatment method for recurrent ventricular tachycardia refractory to invasive treatment methods. The single-fraction delivery of 25 Gy was assumed to produce fibrosis, similar to a post-radiofrequency ablation scar. However, the dynamics of clinical response and recent preclinical findings suggest a possible different mechanism. The data on histopathological presentation of post-radiotherapy hearts is scarce, and the authors provide significantly different conclusions. In this article, we present unique data on histopathological examination of a heart explanted from a patient who had a persistent anti-arrhythmic response that lasted almost a year, until a heart failure exacerbation caused a necessity of a heart transplant. Despite a complete treatment response, there was no homogenous transmural fibrosis in the irradiated region, and the overall presentation of the heart was similar to other transplanted hearts of patients with advanced heart failure. In conclusion, our findings support the theorem of functional changes as a source of the anti-arrhythmic mechanism of radiotherapy and show that durable treatment response can be achieved in absence of transmural fibrosis of the irradiated myocardium.

## Introduction

Despite a decade of increasing clinical experience, and a growing interest in the application of STereotactic Arrhythmia Radioablation (STAR) (1), also known as Cardiac Stereotactic Body Radiotherapy (Cardiac SBRT), the underlying anti-arrhythmic mechanisms are still a subject of scientific debate. Preclinical animal studies postulated that radiosurgery induces scar homogenization through transmural fibrosis, mirroring radiofrequency catheter ablation (RFA) (2). It was never clear, however, whether 25 Gy is capable of inducing such significant structural changes in the human heart (3). The *in vivo* dynamics of treatment response (4) suggested that the anti-arrhythmic effect precedes clinically significant fibrosis. Finally, a recent study by Zhang et al. found that the clinical effects of STAR might be solely associated with functional electrical conduction changes in the myocardium (5).

Kiani et al. were the first to report on human heart pathology changes after STAR and found that even with relatively long follow-up, despite signs of cell death and injury, there is limited fibrosis (6). In a later study by Kautzner et al., the authors supported the pre-clinical theorem of myocardial apoptosis (up to three months post-STAR) followed by a creation of fibrotic lesion (six to nine months post-STAR) in the irradiated region (7). Zhang et al. suggested that the visible fibrosis is likely a consequence of primary heart disease, previously received treatments and that the intensity of fibrosis is not significantly different from what would be observed pre-treatment (5).

Significant differences in the description of post-STAR organ pathology (5–7), but most important clinically relevant differences in efficacy described by the authors (1) could be associated with individual radiosensitivity. The irradiated regions of the myocardium are initially subjected to cardiomyopathies of ischemic and non-ischemic origin and invasive treatment methods (i.e., RFA, ventricular assist device). There is limited data on human healthy myocardium response to radiation, let alone that of the injured heart muscle. It is possible that the initial condition of the tissue determines the sensitivity to radiation, and thus, the rate and durability of clinical response to STAR. In this article, we present the results of a histopathological examination of an explanted heart in a patient with a durable anti-arrhythmic response to irradiation despite a lack of homogenous scar formation, supporting the theorem of non-fibrotic anti-arrhythmic mechanism of STAR.

## Materials and Methods

### Medical History of the Patient

**FIGURE 1:**
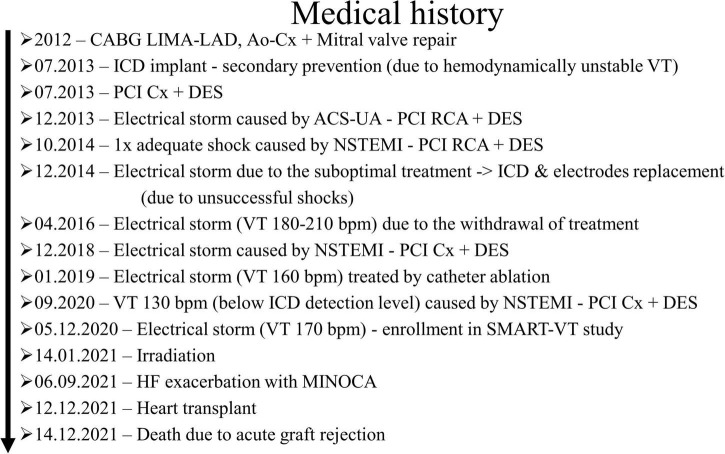
Medical history of the patient treated with STereotactic Arrhythmia Radioablation (STAR). CABG, coronary artery bypass graft surgery; LIMA-LAD, left internal mammary artery – left anterior descending coronary artery; Ao, aortic root; Cx, circumflex branch of the left coronary artery; ICD, implantable cardioverter defibrillator; VT, ventricular tachycardia; PCI, percutaneous coronary intervention; ACS-UA, acute coronary syndrome – unstable angina; DES, drug eluting stent; RCA – right coronary artery; NSTEMI, non-ST-elevation myocardial infarction; MINOCA, myocardial infarction with non-obstructive coronary arteries.

The patient was a 51-year-old overweight male with a medical history of coronary artery disease, heart failure with reduced ejection fraction, recurrent sustained VT, psoriasis, and hyperthyroidism. He underwent coronary artery bypass graft surgery (LIMA-LAD, Ao-Cx), mitral valve replacement in 2012, and was provided with an ICD in secondary prevention of sudden cardiac death in 2013. The patient underwent five percutaneous coronary interventions (PCI) with implantation of stents within the circumflex branch of the left coronary artery thrice and right coronary artery twice between 2013 and 2020. The patient’s medical history is presented in Figure 1.

After the ICD implant, the patient experienced eight hospitalizations due to VTs in total, with different VT morphologies recorded during the patient’s history. Four hospitalizations were associated with acute coronary syndromes with subsequent PCI’s, two episodes happened due to sub-optimal treatment. On two occasions, recurrence of VT happened despite optimal pharmacotherapy and revascularisation. The first such recurrence (01.2019) was followed by a single endocardial RFA of the arrhythmic substrate – the ablated late potentials (LP) were located in the low-voltage zone in the lateral and posterior-septal walls of the left ventricle. The second recurrence (12.2020) developed despite previously introduced oral amiodarone treatment, which led to enrollment in the SMART-VT trial (01.2021). The last episode was not documented on the 12-lead ECG, as the patient received 97 anti-tachycardia pacing bursts and six electrical shocks. In effect, sinus rhythm was present on admission. The electrical storm was caused by a monomorphic VT at 170 bpm (353 ms interval, as per ICD memory). The trial is ongoing and registered under the ClinicalTrials.gov identifier of NCT04642963. A thorough description of the trial protocol can be found in a previously published article (8).

On enrollment, left ventricle ejection fraction (LVEF) was 25% and remained stable at three months after RT. Cardiac ischemia markers remained stable after irradiation and during follow-up. There were no further episodes of sustained VT after STAR. At eight months, the patient was hospitalized due to myocardial infarction with non-obstructive coronary arteries (MINOCA), and subsequent heart failure (HF) exacerbation (LVEF = 10%), which resolved after conservative treatment. Finally, the patient was admitted to the Internal Medicine department nine months after STAR due to a hepatic failure and HF exacerbation. The patient was placed on the heart transplant list, which was performed 11 months post-STAR. The patient died as a result of acute graft failure due to rejection two days after the procedure.

### Radiotherapy Planning and Delivery

The target volume for STAR was defined based on invasive electroanatomic mapping (EAM) and cardiac-gated contrast-enhanced computed tomography (CT) of the heart. The CT was fused with a 1.5 mm treatment planning CT scan. The imaging for treatment planning and the radiotherapy itself were performed using Deep Inspiration Breath Hold (DIBH) technique to account for respiratory motion, and the Iterative Metal Artifact Reduction algorithm to reduce right ventricle ICD lead interferences. The EAM was indirectly compared with CT, and for scientific purposes, directly registered through the aid of the Slicer3D-based software (9), which is presented on the last page of the Supplementary Material.

**FIGURE 2:**
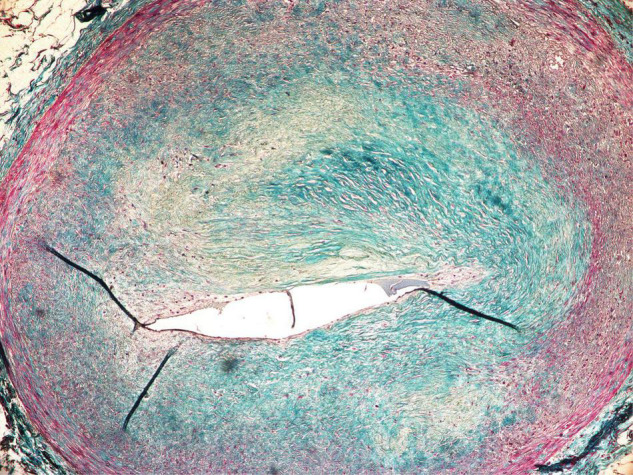
Occluded, atherosclerotic left anterior descending artery with fibrous cap and a lipid core plaque.

The Clinical Target Volume (CTV) measured 42.8 cc and included the whole thickness of the myocardial wall in the region of interest located in the anterolateral part of the left ventricle. The irradiated region was marked by late electrical potentials localized in the akinetic, post-ischemic part of the anterolateral wall of the left ventricle, likely caused by a terminal occlusion of the left anterior descending artery due to advanced atherosclerosis (Figure 2). No prior RFA was performed in the irradiated region. The CTV was expanded by a uniform margin of three mm to account for residual organ motion and positioning uncertainties, resulting in an 88.7 cc Planning Target Volume (PTV).

**FIGURE 3:**
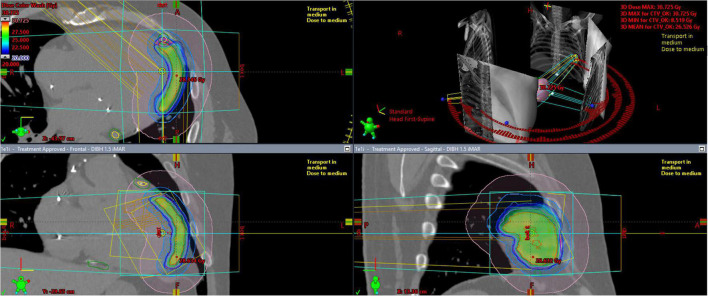
Color-wash representation of the dose distribution within the irradiated heart, ranging from 20 Gy (blue) up to a maximum dose of 30.7 Gy (red). The coronary artery sparing is well-visible in the upper part of the upper-left sagittal projection.

The PTV was irradiated up to a total dose of 25 Gy in one fraction. Sparing of organs at risk had higher priority over delivering a homogenous dose to the whole volume of PTV (Figure 3). The radiotherapy plan was prepared using the Volumetric Modulated Arc Therapy technique and consisted of three arcs. The radiotherapy delivery was performed on a Varian EDGE linear accelerator. The patient positioning verification was performed with DIBH Cone Beam CT (CBCT). The whole treatment session performed with the DIBH technique took 33 min including 18 min for the initial positioning.

### Methodology of the Histopathological Examination

**FIGURE 4:**
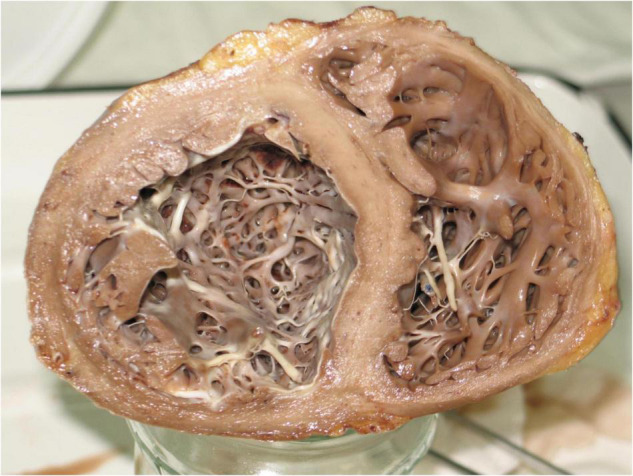
Explanted heart of a patient with early and durable response to STereotactic Arrhythmia Radioablation.

The patient’s heart was acquired after the heart transplantation was performed due to advanced heart failure. The organ was fixed in buffered neutral 4% formalin solution, sectioned, measured, and described (Figure 4). Excised myocardial fragments were dehydrated through graded alcohol and xylene, and embedded in paraffin.

**FIGURE 5:**
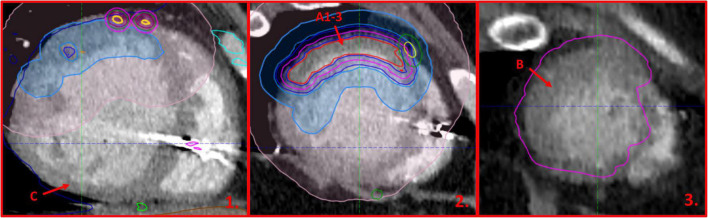
Cardiac-CT images adjusted to the slices performed during the autopsy of the explanted heart. Blue outline (1-2) marks the region irradiated with approximately 7.5 Gy, while violet outline (2-3) shows the planning target volume to which 25 Gy was prescribed. The arrows and letters indicate the approximate localization of the pathology specimens referenced in the article.

A series of four routine samples from both ventricles and 17 further samples from regions of interest were taken by the pathologist (JN). The acquisition of the samples was directed by the attending radiation oncologist (MM) and two cardiologists (MS and MC) with the aid of pre-treatment cardiac CT fused with radiotherapy-planning structures, adjusted to match the geometry of the following slices through the specimen (Figure 5).

The specimens were sectioned into four μm slices and processed using Hematoxylin & Eosin (H&Es), and Masson’s trichrome (MTs) staining. The H&Es were used to examine for the presence of necrosis, inflammation, and vascular changes, while MT was primarily used to demonstrate fibrosis. The histopathological findings were provided with matching microscopic images. All of the available specimens were later scanned to produce panoramic images. The authors are willing to share this data, as described in the Supplementary Material.

## Results

### Electrophysiological Examination

There were no significant changes to QRS duration and morphology over the course of follow-up. The QRS length was initially 140.7 ms, followed by 141 and 138 ms at three and six months, respectively. The patient remained VT-free until the end of the follow-up, and the post-mortem ICD interrogation confirmed that the patient did not experience any non-sustained or sustained VT episodes since STAR up to the heart transplant.

### Gross Examination

The heart was grossly enlarged, measuring approximately 10 × 14 × 9 cm. The epicardial surface was covered by thin opaque fibrin. The site of irradiation macroscopically corresponded with focally thickened fibrosing endocardium and small parietal thrombi inside the left ventricle. The endocardial layer was thick and fibrous. Mean right ventricular thickness varied between five and six mm, whereas dilated left ventricular thickness never exceeded 10 mm.

### Histopathological Examination

**FIGURE 6:**
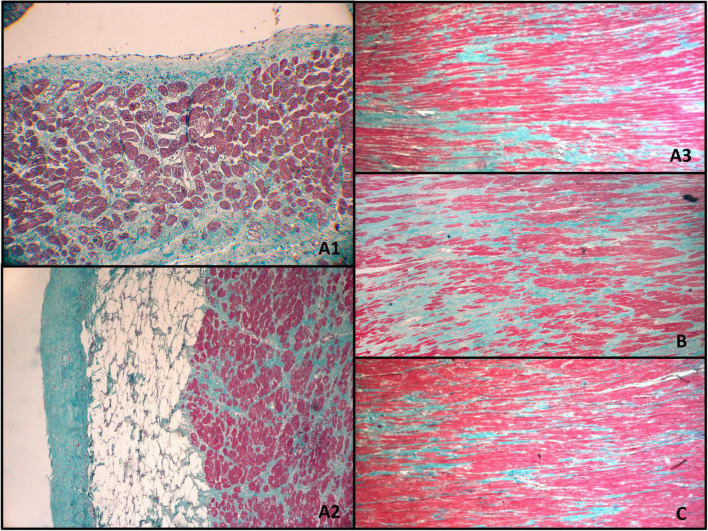
Myocardium 12 months after transmural irradiation with a single dose of 25 Gy. The letters indicate the localization of the specimens as shown in Figure 4. Figures **(A1–A3,B)** were located within the PTV and received approximately 25 Gy, while **(C)** was taken from a non-irradiated region. **(A1)** Endocardium; **(A2)** epicardium; **(A3)** middle part of the myocardium; **(B)** middle part of the myocardium; **(C)** middle part of the myocardium (non-irradiated region).

The myocardium within the irradiated region (Figures 6A1–A3,B) presented with multifocal, mosaic-like fibrosis, and neovascularization of intramuscular connective tissue scars. There were no visible signs of active inflammatory infiltration. The non-homogenous fibrosis was slightly more pronounced sub-endocardial, but there was no transmural scar. The pathological features of the irradiated myocardium were significantly different from the expected sequelae observed after RFA.

Considering that the irradiation was performed in the previously ischemic part of the myocardium, the fibrosis could have been associated with pre-existing ischemic damage to the heart. Moreover, a different etiology of the fibrosis is also suggested by the fact that some of the non-irradiated or marginally irradiated regions presented a similar pattern of fibrosis, albeit subjectively less intense, compared to fully irradiated regions (Figure 6C).

**FIGURE 7:**
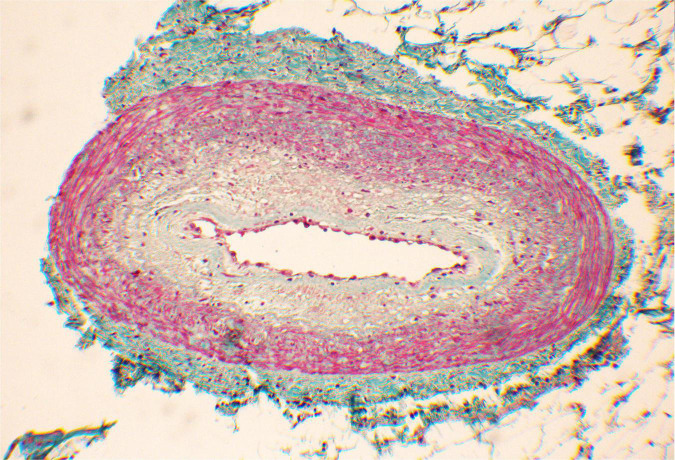
Subendothelial fibrosis of epicardial coronary artery found within the irradiated region.

The small arteries within the region irradiated with 25 Gy presented with sub-intimal fibrosis, and symptoms of intimal changes such as endothelial cell enlargement and the presence of scattered lymphocytes inside thickened intima. The endothelial layer remained intact, and the lumen was preserved (Figure 7).

### Summary of the Pathology Review

Compared to other explanted hearts, the pathological image of the specimen closely resembled those of ischemic cardiomyopathy, and it would not be feasible to determine that this patient had undergone STAR based on pathology examination alone. There was no aneurysm, transmural fibrosis, or resorption of necrosis. The post-STAR region was significantly different from the pre-existing post-RFA scar.

## Discussion

The STAR should not be considered a competitive option for catheter ablation which remains a gold standard in the invasive management of ventricular tachycardia (10). However, considering that several pivotal limitations of RFA do not apply to STAR, the introduction of stereotactic radiotherapy to cardiology and electrophysiology could lead to a substantial improvement in the care of VT patients, and possibly other types of arrhythmias such as atrial fibrillation (11). For example, intramural and epicardial arrhythmic substrate locations, which account for the majority of acute failures in catheter ablation (12), are easily accessible with STAR as the treatment is transmural. Similarly, STAR might be capable of safely ablating volumes adjacent to the critical structures, such as coronary arteries. More data is necessary, but if proven to be correct, this could help to overcome the problem of complex anatomy limiting the application of epicardial ablations.

The initial concept assumed that the biological mechanism of STAR would be similar to RFA through the development of transmural fibrosis in the region of the arrhythmic substrate, and subsequent cessation of electric signal propagation. The clinical experience, however, indicates that STAR induces clinical effects significantly earlier. For example, there are reports of successful electrical storm cessation after STAR (13, 14). Moreover, Kiani et al. found significantly less fibrosis than expected based on the pre-clinical assumptions, despite up to 250 days of follow-up in a total of four explanted hearts (6). As mentioned earlier, a possible game-changing finding was recently published by Zhang et al. (5), suggesting functional changes as a primary mechanism of STAR. If proven to be durable, a significantly different mechanism of treatment could help overcome RFA recurrences. Most importantly this implies a possibility of multimodality treatment, as the irradiated cardiac tissue remains functional and likely sensitive to complementary treatments. This puts into question the use of the word “ablation” when referring to cardiac radiosurgery. As more evidence emerges, it might be more accurate to use the aforementioned “Cardiac SBRT” term, which does not imply bluntly destroying the tissue, but merely associates stereotactic radiotherapy with heart-focused treatment. Another reason to abstain from the “ablation” term would be the effect on small coronary arteries. Despite the expected anti-vascular effect in radiotherapy with fraction doses exceeding 15 Gy (15), we have found that the smaller vessels within the 25 Gy volume remained functional (Figure 7). Although the histopathological examination has shown clear signs of degenerative changes, it is difficult to differentiate between radiation-induced and pre-existing changes, as the patient was suffering from ischemic cardiomyopathy.

The patient described in our study was not considered for epicardial ablation due to anatomical constraints, nor received a left ventricle assist device, both of which can lead to significant scarring, hemorrhage, and edema (16, 17) reported by previous authors (6, 7). The lack of visible necrosis could also be associated with the modality of heart rhythm cessation. The patient underwent cardioplegia with isotonic and osmotic solution, which lowers the metabolism and prevents degenerative changes and cell death in cardiomyocytes. Cardiopulmonary resuscitation and postmortem period in hearts explanted from deceased patients can induce previously described necrosis and cardiomyocyte vacuolization (6, 7).

STereotactic Arrhythmia Radioablation is challenged by significant conceptual variability between centers. The target delineation can be based on non-invasive cardiac mapping (4), invasive EAM (8), or even primarily on 12-lead ECG and medical imaging (18). The transfer process of the EAM data to radiotherapy planning systems ranges from indirect comparison to software-based transformations (9), and the median volume of the final structures varies almost sevenfold between centers (1). Moreover, several different platforms have been used to perform STAR, including C-arm linear accelerators, CyberKnife, and proton beams. To account for the lack of standardization, a European consortium and prospective registry called STOPSTORM was established (19). The registry is funded by a European grant from the Horizon 2020 Framework Programme for Research and Innovation and aims to learn from every case treated in Europe, ultimately resulting in standardized treatment guidelines for STAR.

We acknowledge the limitations of the study, including the case-report nature of the publication and the lack of a control group. It has to be pointed out that theoretically, the patient could have been VT-free without intervention, as there were VT-free periods earlier in the medical history (Figure 1). This would not change the pathological description presented in this article but could affect its clinical implications and conclusions. Considering that STAR is an emerging treatment modality, and heart transplants in such patients are exceptionally rare, we believe that our study provides important data, which might be significant for the development of an accurate biological model of the in-human antiarrhythmic effect of STAR.

## Conclusion

Our findings support the theorem that the anti-arrhythmic effect can occur and be persistent over time despite the lack of RFA-like scar formation. Moreover, the microscopic analysis revealed that up to 12 months after RT, there was no significant occlusion of the small vessels within the high irradiation dose region of the patient’s heart, suggesting that coronary artery sparing might be of less importance than previously assumed by some of the authors.

## Data Availability Statement

The authors are willing to share the raw data supporting the conclusions of this article upon reasonable request, as described in the Supplementary Material.

## Ethics Statement

This study describing a post-mortem histopathological analysis involved human participants, and was a part of the SMART-VT study reviewed and approved by the Bioethics Committee of Maria Skłodowska-Curie National Research Institute of Oncology, Gliwice Branch (KB/430-45/20). The patient provided their written informed consent to participate in the SMART-VT study, and at the time of the treatment provided a separate written informed consent for the pathological examination of the heart in case of heart transplant or death, such as the one described in the article.

## Author Contributions

MM, MS, KG, and SB contributed to the conception and design of the study. JN performed the pathology examination with the aid of MM and MC. MM, MS, JN, and MC organized the database. MM and MS performed the image editing and wrote the first draft of the manuscript. JN and SB wrote sections of the manuscript. MM, MS, MC, JB, TJ, TL, RK, ŁD, WW, ADr, AG, AB, KG, and SB participated in the described treatment of the patient. ADy and MZ contributed to the heart transplant. KK and AK assisted with the data collection and preparation. All authors contributed to the manuscript revision, read, and approved the submitted version.

## Conflict of Interest

The authors declare that the research was conducted in the absence of any commercial or financial relationships that could be construed as a potential conflict of interest.

## Publisher’s Note

All claims expressed in this article are solely those of the authors and do not necessarily represent those of their affiliated organizations, or those of the publisher, the editors and the reviewers. Any product that may be evaluated in this article, or claim that may be made by its manufacturer, is not guaranteed or endorsed by the publisher.

## Supplementary Material

Supplementary Material 1 Visual presentation of the location of directed samples, description of the possibility of sharing panoramic images and example of direct EAM to CT registration.
