# Supplementary material for: Histopathological Examination of an Explanted Heart in a Long-Term Responder to Cardiac Stereotactic Body Radiotherapy (STereotactic Arrhythmia Radioablation)
Source: Front Cardiovasc Med. 2022 Jul 7;9:919823. doi: 10.3389/fcvm.2022.919823 (PMC9302025; doi:10.3389/fcvm.2022.919823)
Supplement: Supplementary Material 1 — Visual presentation of the location of directed samples, description of the possibility of sharing panoramic images and example of direct EAM to CT registration. [file Presentation_1.pptx]

## Slide 1
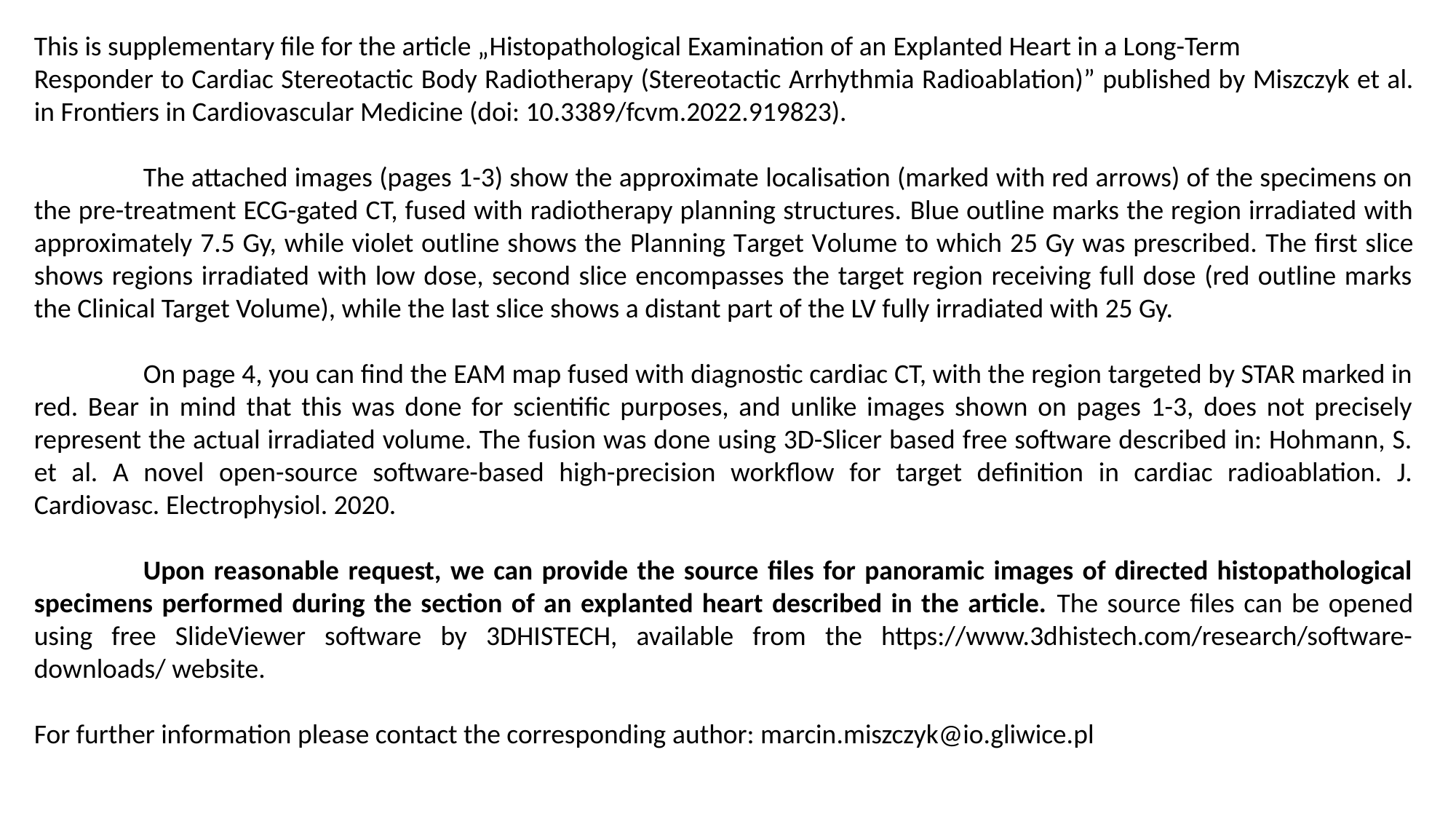

This is supplementary file for the article „Histopathological Examination of an Explanted Heart in a Long-Term
Responder to Cardiac Stereotactic Body Radiotherapy (Stereotactic Arrhythmia Radioablation)” published by Miszczyk et al. in Frontiers in Cardiovascular Medicine (doi: 10.3389/fcvm.2022.919823).
	The attached images (pages 1-3) show the approximate localisation (marked with red arrows) of the specimens on the pre-treatment ECG-gated CT, fused with radiotherapy planning structures. Blue outline marks the region irradiated with approximately 7.5 Gy, while violet outline shows the Planning Target Volume to which 25 Gy was prescribed. The first slice shows regions irradiated with low dose, second slice encompasses the target region receiving full dose (red outline marks the Clinical Target Volume), while the last slice shows a distant part of the LV fully irradiated with 25 Gy.
	On page 4, you can find the EAM map fused with diagnostic cardiac CT, with the region targeted by STAR marked in red. Bear in mind that this was done for scientific purposes, and unlike images shown on pages 1-3, does not precisely represent the actual irradiated volume. The fusion was done using 3D-Slicer based free software described in: Hohmann, S. et al. A novel open-source software-based high-precision workflow for target definition in cardiac radioablation. J. Cardiovasc. Electrophysiol. 2020.
	Upon reasonable request, we can provide the source files for panoramic images of directed histopathological specimens performed during the section of an explanted heart described in the article. The source files can be opened using free SlideViewer software by 3DHISTECH, available from the https://www.3dhistech.com/research/software-downloads/ website.
For further information please contact the corresponding author: marcin.miszczyk@io.gliwice.pl

## Slide 2
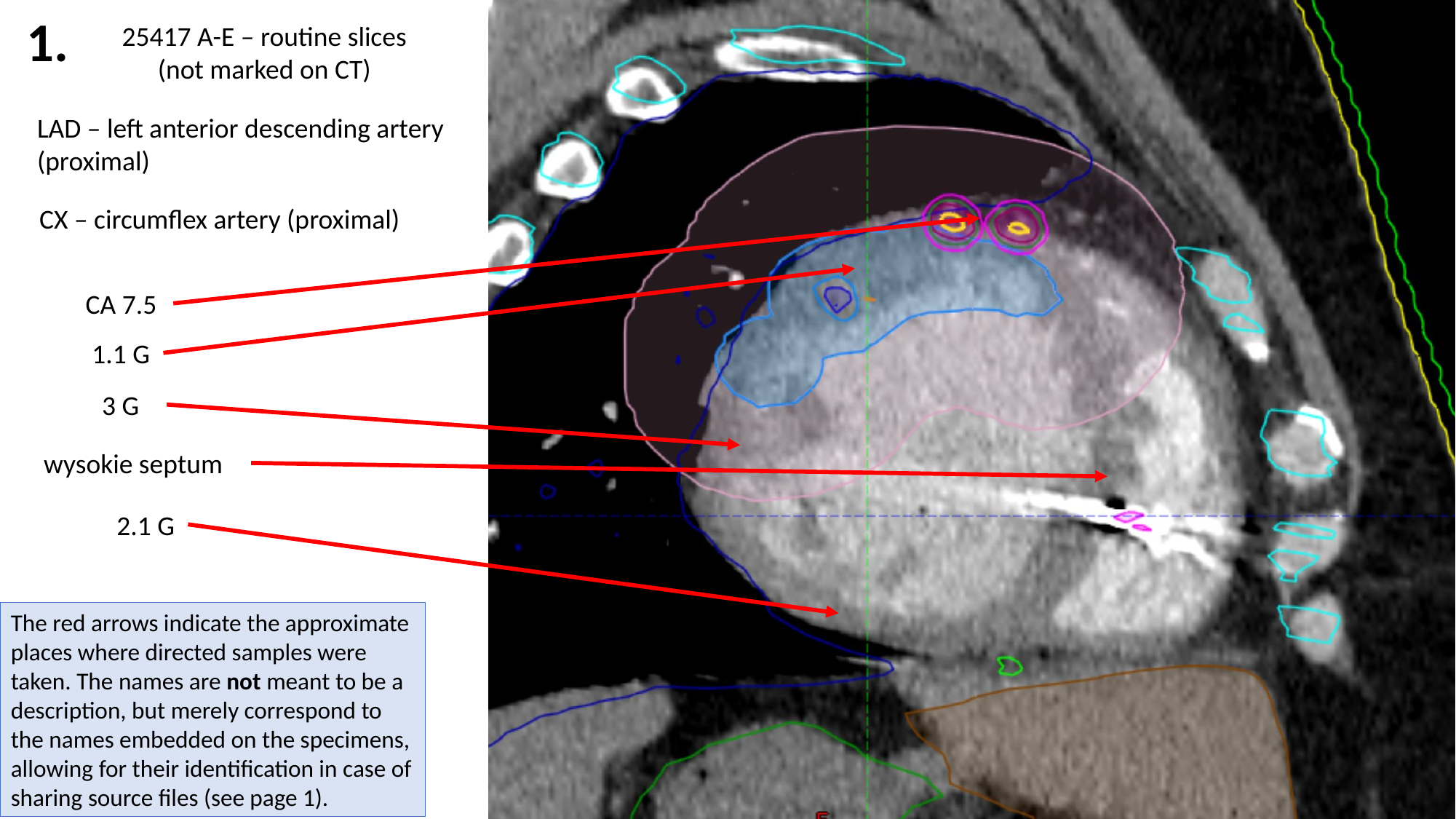

1.
25417 A-E – routine slices
(not marked on CT)
LAD – left anterior descending artery (proximal)
CX – circumflex artery (proximal)
CA 7.5
1.1 G
3 G
wysokie septum
2.1 G
The red arrows indicate the approximate places where directed samples were taken. The names are not meant to be a description, but merely correspond to the names embedded on the specimens, allowing for their identification in case of sharing source files (see page 1).
M Miszczyk, 14/12/2021

## Slide 3
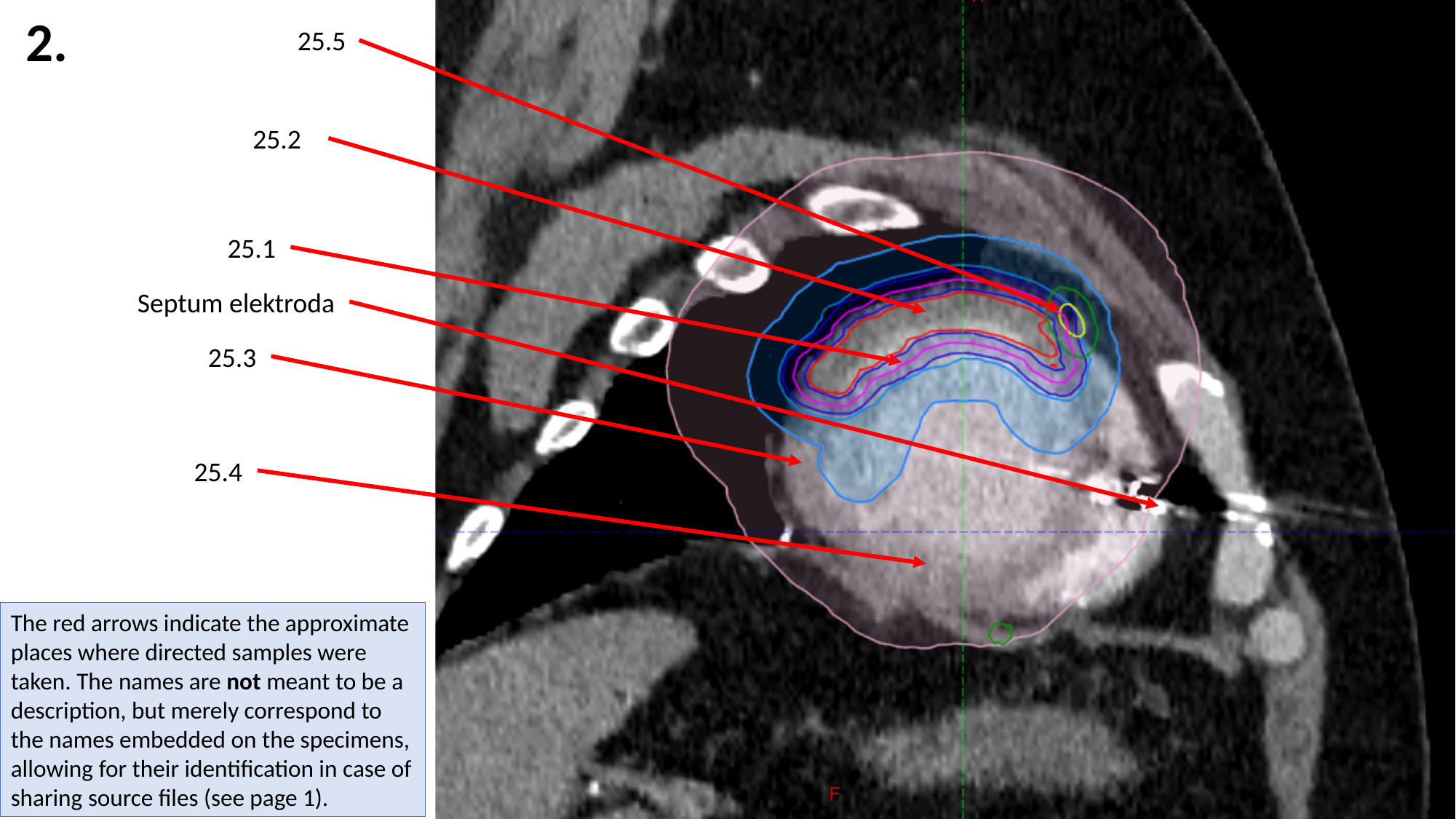

2.
25.5
25.2
25.1
Septum elektroda
25.3
25.4
The red arrows indicate the approximate places where directed samples were taken. The names are not meant to be a description, but merely correspond to the names embedded on the specimens, allowing for their identification in case of sharing source files (see page 1).
M Miszczyk, 14/12/2021

## Slide 4
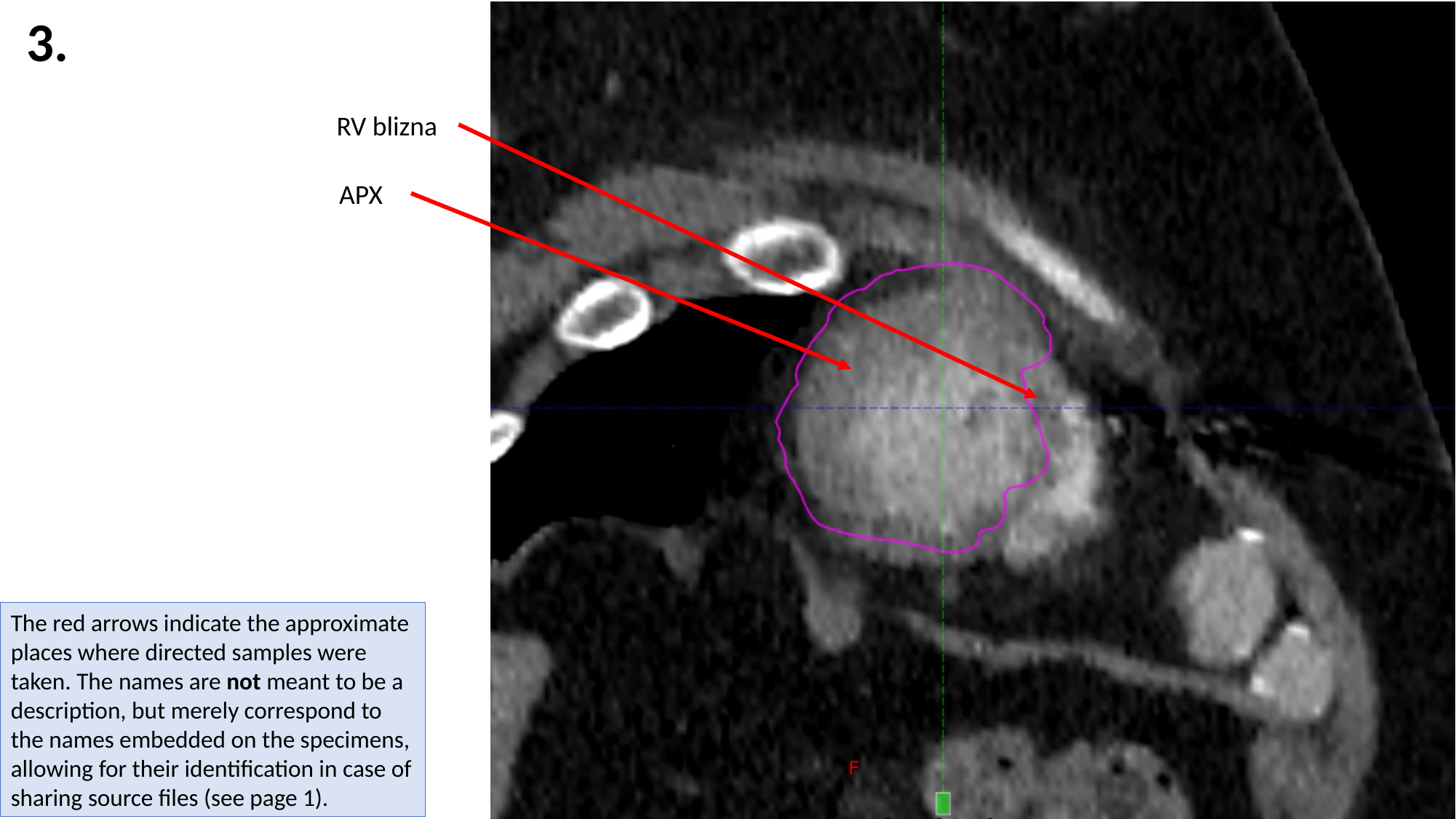

3.
RV blizna
APX
The red arrows indicate the approximate places where directed samples were taken. The names are not meant to be a description, but merely correspond to the names embedded on the specimens, allowing for their identification in case of sharing source files (see page 1).
M Miszczyk, 14/12/2021

## Slide 5
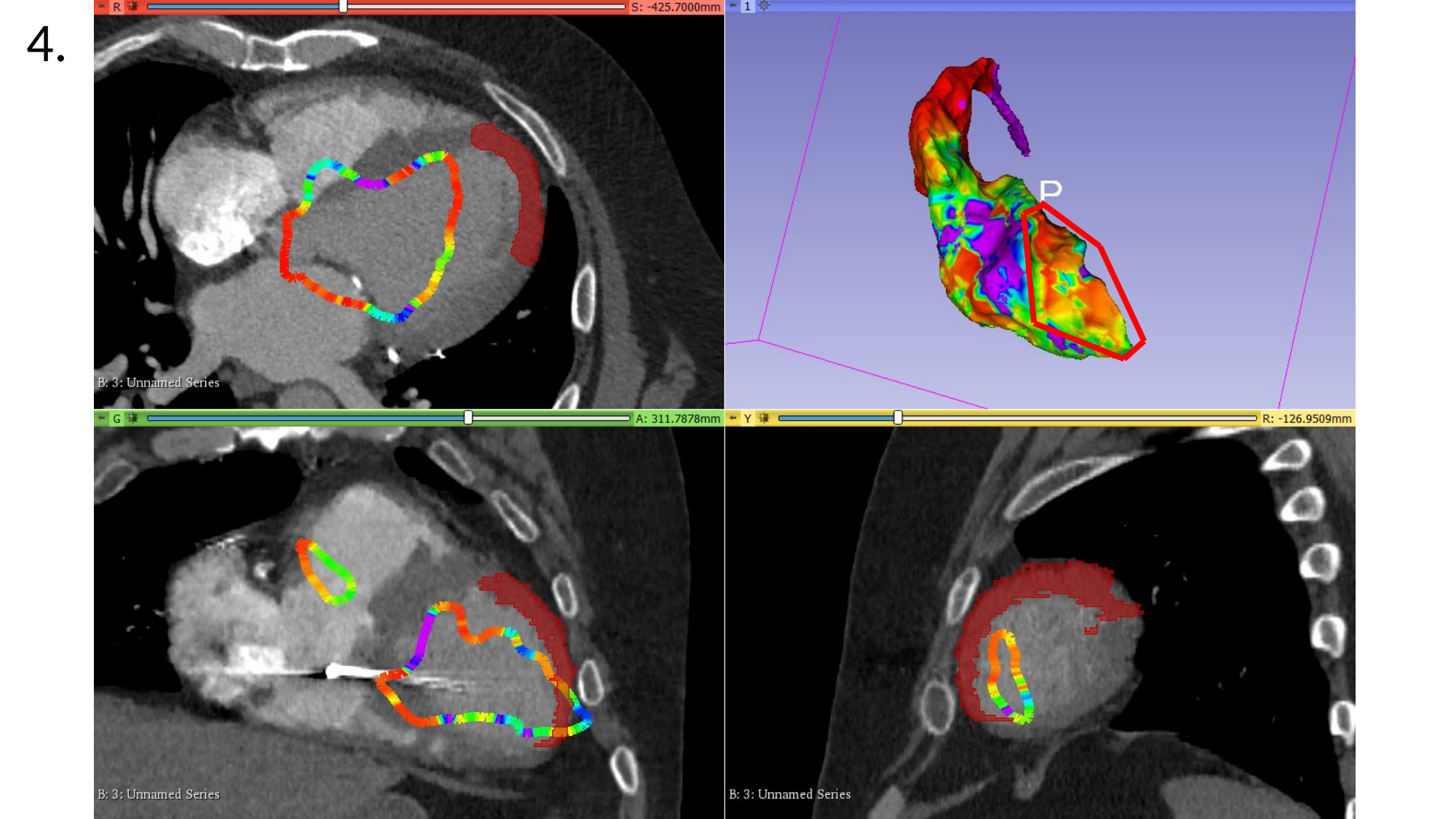

4.
